# Nutrigenomics of Dietary Lipids

**DOI:** 10.3390/antiox10070994

**Published:** 2021-06-22

**Authors:** Laura Bordoni, Irene Petracci, Fanrui Zhao, Weihong Min, Elisa Pierella, Taís Silveira Assmann, J Alfredo Martinez, Rosita Gabbianelli

**Affiliations:** 1 Unit of Molecular Biology and Nutrigenomics, School of Pharmacy, University of Camerino, 62032 Camerino, Italy; laura.bordoni@unicam.it; 2 School of Advanced Studies, University of Camerino, 62032 Camerino, Italy; irene.petracci@unicam.it (I.P.); fanrui.zhao@unicam.it (F.Z.); 3 College of Food Science and Engineering, Jilin Agricultural University, Changchun 130118, China; minwh2000@jlau.edu.cn; 4 School of Medicine, Faculty of Clinical and Biomedical Sciences, University of Central, Lancashire, Preston PR1 2HE, UK; EPierella1@uclan.ac.uk; 5 Postgraduate Program in Medical Sciences: Endocrinology, Faculty of Medicine, Federal University of Rio Grande do Sul, Porto Alegre 90035093, Brazil; taisassmann@hotmail.com; 6 CIBERobn Fisiopatología de la Obesidad, Instituto Carlos III, Precision Nutrition Program IMDEA Food UAM-CSIC, 28049 Madrid, Spain; jalfmtz@unav.es

**Keywords:** nutrigenomics, fatty acids, inflammation, gut microbiota, personalized nutrition

## Abstract

Dietary lipids have a major role in nutrition, not only for their fuel value, but also as essential and bioactive nutrients. This narrative review aims to describe the current evidence on nutrigenomic effects of dietary lipids. Firstly, the different chemical and biological properties of fatty acids contained both in plant- and animal-based food are illustrated. A description of lipid bioavailability, bioaccessibility, and lipotoxicity is provided, together with an overview of the modulatory role of lipids as pro- or anti-inflammatory agents. Current findings concerning the metabolic impact of lipids on gene expression, epigenome, and gut microbiome in animal and human studies are summarized. Finally, the effect of the individual’s genetic make-up on lipid metabolism is described. The main goal is to provide an overview about the interaction between dietary lipids and the genome, by identifying and discussing recent scientific evidence, recognizing strengths and weaknesses, to address future investigations and fill the gaps in the current knowledge on metabolic impact of dietary fats on health.

## 1. Introduction

Diet is one of the most important environmental factors able to maintain health and prevent diseases, starting from early neonatal life with a myriad of nutritional properties, affecting energy supply, regulatory functions, and structural roles [1,2]. During recent last years, omics approaches, including genetics, epigenetics, and gut microbiome analyses, have been introduced in nutrition research, providing a comprehensive picture able to renovate molecular nutrition studies toward an advanced and systemic vision. Food has been studied not only from a chemical prospective, but also for the capacity of metabolites produced by food oxidation to modulate gene expression, directly (nutrigenomics) or by epigenetics remodeling (nutri-epigenomics) [3]. However, inter-individual differences (i.e., the genetic variability) and environmental exposures (i.e., physical activity, drug, food pesticide residues, etc.) contribute to produce a plethora of multifaceted effects [4]. For these reasons, understanding molecular effects of food on human health is an ambitious but promising goal, which could strongly impact dietary choices considering not only food composition, but also its nutrigenomic and nutri-epigenomic properties. 

In particular, one of the aims of nutrition research is to control systemic chronic inflammation (SCI), that negatively affects human health [5]. Since, SCI can increase the risk of developing several diseases (i.e., metabolic syndrome, cardiovascular disease, neurodegeneration, cancer, etc.), one major focus of nutrigenomics is to identify those foods or food-derived metabolites that can modulate pro and anti-inflammatory genes expression. In this context, dietary lipids can exert both pro- and anti-inflammatory effects, according to their chemistry. Unfortunately, consumers’ dietary unhealthy habits frequently results in a poor nutrition rich in ultra-processed food intake. However, we have a partial knowledge of the composition of the food we daily eat [6], and of the impact that food can have on our individual genome, several findings about nutrigenomic properties of dietary lipids from both humans and animal models have been collected. 

In this milieu, the objectives of the present narrative review are framed as follows: (1) describe the lipid content in plant and animal-based food products; (2) outline the molecular mechanisms associated to the pro- and anti-inflammatory responses of dietary fatty acids; (3) summarize the main findings on nutrigenomics of dietary lipids acquired from animal and human studies; (4) explain the mechanisms implicating dietary lipids in the modulation of the gut microbiota compositions and metabolites production; (5) discuss the main impact of dietary lipids on human health. 

The search strategy includes the terms “nutrigenomics AND dietary lipids” or “fats AND animal-based food products” or “fats AND plant-based food products” or “fatty acids AND inflammation” or “fatty acids AND human health” or “dietary lipids AND gut microbiota” or “fats AND gut metabolites” as keywords. The search has been performed in the NCBI-PubMed database. The records have been screened for relevant information and only papers published within the period 2010–2020 have been selected and studied. 

## 2. Dietary Lipids: Fatty Acids in Plant- and Animal-Based Food Products

Dietary lipids’ main roles are to supply energy, provide essential fatty acids, guarantee the required lipids for cellular structures (i.e., membranes), regulate signaling systems, and ease the absorption of fat-soluble vitamins (A, D, E and K). According to their physicochemical structure, dietary lipids exert a different nutrigenomic impact, modulating the expression of genes involved in the control of inflammation, blood pressure, lipid metabolism, and other metabolic responses [7,8,9].

**Figure 1:**
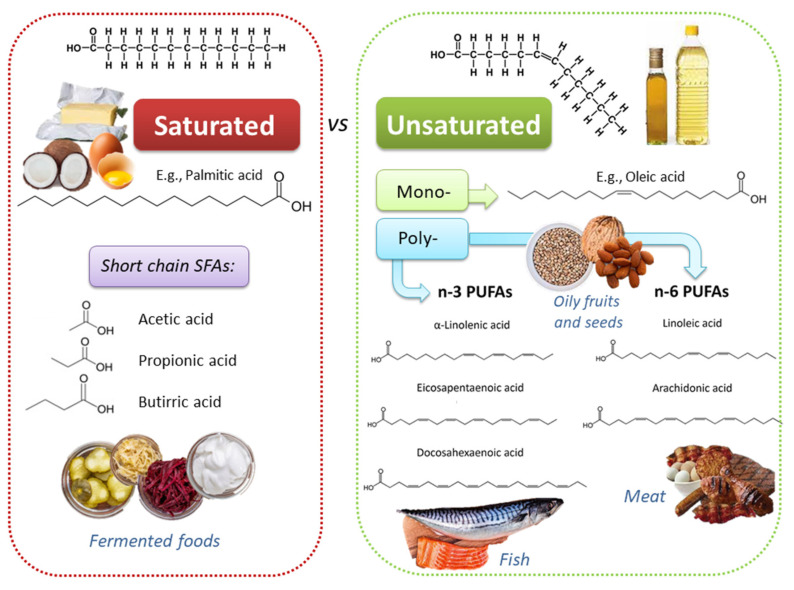
Chemically diverse lipid sources, with heterogeneous physico-chemical and biological properties, can be found in different foods.

Dietary lipids comprise fatty acids (FAs) and cholesterol. FAs can be free (i.e., butter, oils, milk) or esterified in food to form triacylglycerols and other complex lipids (i.e., phospholipids, glycolipids, and glycoproteins). FAs differ for the length of the carbon chains, which influence the intimate physicochemical properties, and for the absence or the presence of one or more double bonds; based on this second feature, three main classes exist: (i) saturated FAs (SFAs), such as palmitic acid, myristic acid, and lauric acid; (ii) monounsaturated FAs (MUFAs), such as ω-9 oleic acid (OA); (iii) polyunsaturated FAs (PUFAs) (Figure 1). PUFAs include two essential groups, omega-3 (or n-3 or ω-3) and omega-6 (or n-6 or ω-6) FAs, which have to be obtained through the diet because they cannot be synthetized by human enzymes. Among ω-3 FAs, linolenic acid (ALA; 18:3 ω-3) is mostly contained in plants (i.e., flaxseed, canola, soybean, nuts, walnuts, chia seeds, etc.), while eicosapentaenoic acid (EPA; 20:5 ω-3) and docosahexaenoic acid (DHA; 22:6 ω-3) are present mostly in fish, seafood, and marine algae. Among ω-6 FAs, linoleic acid (LA; 18:2 ω-6) is provided by seed oils, soybean, nuts, and cereals, while arachidonic acid (ARA; 20:4 ω-6) is found in poultry and eggs [10]. Land-based food chain is dominated by higher LA than ALA; these two essential FAs give rise to ω-6 and ω-3 series, which have a different impact on the inflammatory response.

Various SFAs are supplied by both animal- (i.e., meat, seafood, milk, cheese, eggs, etc.) and plant-based food (i.e., oilseeds, olives, avocados, walnuts, etc.). Meat content in SFAs depends on animal species, growth, and environmental conditions; generally, SFAs content decreases going from beef (6.2 g/100 g) to pork (5.4 g/100 g) and to chicken breast (1.4 g/100 g) [11]. Meat content in MUFAs (i.e., ω-3) is very low (0.1–0.2 g/100 g), while the ω-6 LA is 2.9, 3.2, and 0.8 (g/100 g) in beef, pork, and chicken breast, respectively [11]. However, feed background of ruminants (i.e., forage- or grass-fed containing high amount of ALA) significantly contributes to increase the concentration of ω-3 FAs in red meat [12]. Among animal-based food products, whole milk is rich in SFAs (2.2 g/100 g), whose content decreases in skimmed milk (0.1 g/100 g). Butter is an animal fat containing a large amount of SFAs (52.9 g/100 g) and cholesterol (227 mg/100 g) [11,13]; margarine, obtained from partially hydrogenated vegetable oils, includes less SFAs (8.5 g/100 g) than butter, but it is rich in trans-FAs, which increase the risk to develop cardiovascular and neuronal disorders [14,15,16].

Among the most commonly used edible oils, coconut oil (CO) contains the highest SFAs content (81.2–94%), followed by palm oil (PO) (49.7–57.5%), soybean oil (6.0–24.0%), olive oil (12.5–20.9%), corn oil (15–16%), walnut oil (5–17%), and sunflower oil (9–13%) [17]. The content in the OA, an ω-9 MUFAs, (which exerts anti-inflammatory properties and improve blood pressure [18,19,20,21]) decreases form olive oil (54.5–80.2%), to PO (37.3–40.8%), corn oil (27.6–34.6%), soybean oil (15–36%), sunflower oil (16.4–27.6%, walnut oil (10–20%) and CO (5–10%). LA concentration increases starting from coconut oil (1–2.5%), to PA (9.1–11%), olive oil (4.9–21.2%), corn oil (48.8–55.3%), soybean oil (42.8–56.1%), walnut oil (55–70%), and sunflower oil (60.2–72.1%). ALA content decreases from walnut oil (10–18%) to soybean oil (2–14%), CO (0.2–2.5%), sunflower oil (0.07–1.8%), olive oil (0.7–1.5%), corn oil (0.6–1.49%), and PA (0.01–0.25%) [17]. 

Other aspects should be considered in the evaluation of the nutrigenomic properties of plant- and animal-based fats: (i) the presence of bioactive compounds that contribute to modulate gene expression; (ii) the oil oxidative stability. For example, olive oil contains a large number of bioactive substances, such as polar phenolic substances (i.e., hydroxytyrosol and derivatives like oleuropein complex, tyrosol) and α-tocopherol, which plays a key role in the health promotion and oil stability [22]. Another example is the thymoquinone, contained in *Nigella sativa* oil, which contributes to modulate the noteworthy anti-inflammatory properties of this oil, which resulted to be quite similar to olive oil in low-grade inflammation models [23,24,25]. To increase their healthy properties, some foods have been also fortified with ω-3 PUFAs (i.e., egg sticks, hen eggs, dahi, chicken-meat, milk, etc.) [26,27,28,29,30]. Moreover, research has identified also other kinds of vegetable oils that could represent a suitable source of healthy lipids. These includes oils derived from cucumber, tomato, pumpkin or carrot seeds, which contain different profiles of FAs; for instance, OA is largely contained in carrot seed oil (78.97 g/100 g of total FAs) [31].

Another significant aspect to consider is the oxidative status of oils and of the other dietary lipids, which depends on their unsaturated FAs content (i.e., the presence of double bonds is associated to a faster oxidation rate). For example, LA, which has two double bonds, shows an oxidation rate ten times higher than OA, which has only one double bond [32]. Hydroperoxides and malondialdehyde (MDA) can be produced during technological processing, light exposure, oxygen, and temperature storage, as well as due to cooking; lipid oxidation decreases the quality of dietary FAs and their chemical, sensory and structural properties (i.e., membrane phospholipids) [33,34,35]. For all these reasons, vegetal- and animal-based food should be evaluated also considering both intrinsic components (i.e., FAs composition, antioxidants, water, etc.) and external factors (i.e., light, temperature, oxygen), which contribute to preserve their original naïve composition. For example, the high quality of olive oil, with a lower oxidative index in respect to others [32], depends on the high concentration of MUFAs and the low PUFAs content; this is an additional value over and above the bioactive substances contained in the unsaponifiable fraction, which also has a well-balanced saponifiable fraction, rich in OA and poor in SFAs. 

Considering the metabolic and functional roles of dietary lipids, from 20 to 35% of the total energy should be obtained from fat. For each type of fat, the recommendations are as follows: SFAs < 10%, PUFAs 5–10%, of which ω-6 PUFAs 4–8% and ω-3 PUFAs 0.5–2%, trans-FAs < 1% and cholesterol < 300 mg, according to accepted dietary recommendations [36].

In summary, plant- and animal-based foods contribute to modulate molecular responses according to their chemical composition and stability; whether prefer plant- or animal-based food has been the object of study for a long time, and research on large cohorts of middle-age adults supports the positive impact of a higher adherence to a plant-based diet due to the presence of bioactive compounds able to reduce the risk of mortality (i.e., cardiovascular disease mortality (−19%), all-cause mortality (−11%)) [37]. Multiple studies in different models (i.e., cell lines, preclinical and human studies) have provided a wealth of information on the molecular basis of dietary lipids’ impact on gene expression. Henceforth, the following sections describe these data in order to give a comprehensive picture of the nutrigenomic impact of dietary lipids.

## 3. Dietary Lipids Bioavailability, Bioaccessibility, and Toxicity

**Figure 2:**
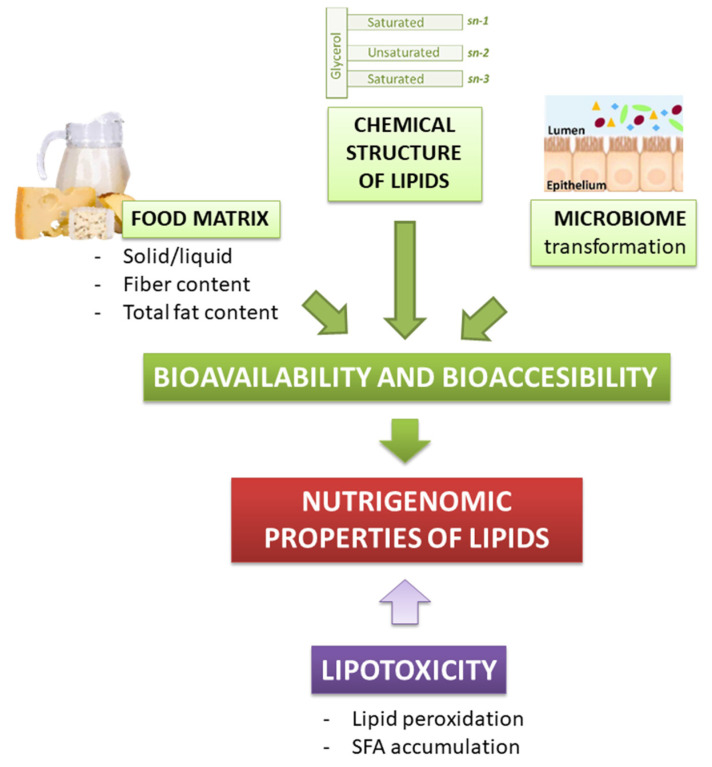
Dietary lipids bioavailability, bioaccessibility, and lipotoxicity contribute to their nutrigenomic properties.

As well as for other bioactive food compounds, the nutritional effect of lipids depends on their release in the gastrointestinal tract, intestinal absorption, and metabolism (i.e., their bioaccessibility and bioavailability). Lipids bioavailability refers to the fraction of the ingested lipids that eventually reaches the systemic circulation, to be later distributed to tissues and organs, where it may be either stored, utilized, or excreted. However, not the whole lipid cargo of a meal is bioavailable to exert a biological function. Indeed, only the bio-accessible lipid fraction, which is the one released from the food matrix, is available for intestinal absorption. Hence, for lipids, the possibility to exert a biological effect is strictly dependent on their bio-accessibility (Figure 2) [38].

The interaction between dietary fats and the food matrix (i.e., food composition) is very important to guarantee their accessibility, digestibility, and bioavailability. Martínez-Ramírez et al. described how the digestibility of FAs is influenced by lipid source and content of a meal, dietary fibers, host animals, and other molecules (e.g., mucilage and cyanogenic glycosides in flaxseed) [39]. For instance, it is well documented that the digestibility of lipids inversely correlates with the dietary fiber content of a meal [40]. Also, the total fat content of a meal can modulate the bioavailability of dietary FAs, mainly through the stimulation of pancreatic juice secretion and the activation of pancreatic lipases, which makes dietary FAs more accessible. The role of the meal fat content is especially crucial for essential ω-3 PUFAs, in particular for dietary ALA, whose conversion rate into physiologically active EPA and DHA is very limited: even though data from literature may slightly differ, approximately 5% of ALA is converted to EPA and less than 0.5% to DHA [41]. Moreover, it was already known that the conversion rate is dependent on certain physiological states and on the concentration of ω-6 PUFAs or other fats [42]. In particular, previous evidence suggested that with a background diet rich in saturated fats, the conversion of ALA to EPA is around 6%, while approximately 3.8% ALA is converted to DHA. Instead, with a background diet rich in ω-6 PUFAs, this conversion decreases by 40 to 50% [43]. However, ALA conversion seems to be unaffected by the ω-6/ω-3 ratio, and being dependent only on their absolute amounts in the diet [42]. Lawson and Hughes showed that ω-3 PUFAs absorption rate can be increased three-fold when co-ingested with a high-fat meal relative to a low-fat meal [44,45]. Interestingly, the ECLIPSE studies [Epanova^®^ Compared to Lovaza^®^ In a Pharmacokinetic, Single-dose, Evaluation] have proved that a novel free FAs (FFAs) formulation for ω-3 supplements (OM-3 FFA) exhibits a 4-fold greater bioavailability in overweight patients either in a single dose (4 g) [46,47] or over a two-week period under low-fat dietary intake [48]. This might help in subjects with hypertriglyceridemia that need to maximize the intestinal absorption of EPA and DHA, while maintaining their total dietary fat intake low.

Several studies proved that not only the composition, but also the structure (solid or liquid) of the food matrix is an important modulator of dietary fats accessibility. An in vitro study on microencapsulated tuna oil showed that either the way of ingestion or the chemical formulation strongly affect the release of EPA and DHA from tuna oil during digestion [49]. In vitro digestion of tuna oil in the form of neat microencapsulated tuna oil powder (25% oil loading) or in food matrices (orange juice, yogurt, or cereal bar) fortified with microencapsulated tuna oil powder (fortification was equivalent to 1 g of tuna oil per recommended serving size), showed that the percentage of EPA and DHA released from tuna oil was greater (73.2–78.6%) for neat powder, fortified orange juice, and yogurt compared to the fortified cereal bar (60.3–64.0%), proving that the food matrix, its structure (e.g., solid versus liquid), and its composition (greater fiber content of the cereal bar) may affect in vitro digestion of microencapsulated tuna oil powder [49]. An in vivo study, based on three matrices having the same composition but different structures (DHA-enriched egg in the form of omelet, hard-boiled egg, and mousse) found that the egg in the form of omelet was the most efficient vector for delivering DHA into systemic circulation (33.3 ± 3.6 µg/mL and 17.9 ± 3.7 µg/mL at 10 and 24 h after feeding, respectively), further stressing the impact of food matrix structure on DHA bioavailability [50]. Lastly, differently structured food products can lead to different profiles of postprandial lipemia. For example, for dairy products, Lamothe et al. showed that the fatty acid release in the intestinal phase is faster when the cheese matrix derives from homogenized milk [51]. Also, the cheese’s composition, viscosity, and structure (more or less solid) have an impact on the kinetics of digestion of the lipids from cheeses and can affect the peak of postprandial triglyceridemia [52]. Specifically, the denser and more cohesive the cheese matrix structure, the lower the rate gastric digestion of lipids [53].

In addition, several human studies have focused on the role of the chemical structures of lipids on Fas bioavailability [44]. Over 90% of dietary lipids are esterified to triacylglycerol (TAGs), but they can only be absorbed when released from the TAGs as non-esterified FAs (FFAs) or as 2-monoacylglycerols (2-MAGs) after digestive lipolysis [54]. The rate at which FAs are released from TAGs during lipolysis inversely correlates with their chain length, and also depends on their intramolecular structure on the glycerol backbone (internal sn-2 position, external sn-1 and sn-3 positions). Thus, digestive lipases preferentially hydrolyse FAs esterified on sn-1,3 positions of glycerol backbone, but when TAGs contain long-chain FAs in these positions, their hydrolytic activity decreases. For instance, long-chain saturated FAs, such as palmitic acid and stearic acid, are mainly bound in the sn-1 and sn-3 positions of TAGs, but exhibit poor bioavailability because they tend to form complexes in presence of divalent ions (Ca^2+^ and Mg^2+^), resulting in the formation of insoluble soaps in the small intestine that can be lost in the stools [54,55]. Instead, when PUFAs are esterified in the sn-1 and sn-3 positions of TAGs with double bonds close to the carboxyl group, the hydrolytic activity of lipases decreases because of steric hindrance problems, with an impact on PUFAs bioavailability [54]. Conversely, PUFAs esterified at the sn-2 position of TAGs appear to be more efficiently absorbed. For example, supplementation of human adults with fish oil, which has DHA mostly on sn-2 position and EPA on the sn-1 and sn-3 positions, resulted in more rapid incorporation of DHA in plasma TAGs than EPA [56]. Coherently, a six-month supplementation of identical doses of EPA+DHA resulted in a faster and higher increase in the ω-3 index (i.e., the percentage of EPA+DHA in red blood cell membranes) when consumed in the form of triacylglycerides than as ethyl esters (EEs) [57]. Studies on the kinetics of ω-3 PUFAs confirmed the following trend for bioavailability: FFAs  >  TAGs  >  diacylglycerol (DAGs)\MAGs > EEs [58,59,60], although recent findings [61] question the previously stated greater bioavailability of TAGs compared to EEs [62], while information on the bioavailability of ω-3 PUFAs esterified to phospholipids is so far poor and inconclusive [63,64].

Lastly, PUFAs’ bioavailability and transformation can be also affected by the gut microbiota. In fact, some microbial species, such as *Butyrivibrio fbrisolvens*, *Clostridium proteoclasticum*, or *Lactobacillus plantarum* play a crucial rule in the transformation of ω-6\ω-3 PUFAs into conjugated FAs that may further undergo hydrogenation to saturated FAs, reducing the amount of the bioavailable unsaturated fraction [65].

In addition to bioavailability, another aspect to mention before discussing the molecular effects of lipids is lipotoxicity. Lipotoxicity arises as a consequence of the accumulation of biosynthetic lipid intermediates, such as ceramides and diglycerides, within non-adipose tissues, and induces endoplasmic reticulum stress, inflammation, mitochondrial dysfunction, and ultimately apoptosis [66]. Pancreatic β-cells are among the most affected cells, whose impaired insulin secretion results in the development of insulin resistance, T2DM or other obesity-related pathologies, such as cardiovascular diseases and the metabolic syndrome [67,68,69,70]. Lipotoxicity may also affect other organs, such as kidney, liver, heart and skeletal muscle, and clinical manifestations are renal dysfunction, anemia, heart failure, and sarcopenia [71]. To mitigate the detrimental effects of ectopic lipid accumulation, lipid-lowering drugs supported by suitable dietary changes may be valid tools. In this regard, recent studies have shown that soy proteins and soy isoflavones are able to decrease lipotoxicity by modulating insulin secretion and lowering ceramides accumulation [72,73]. Another mechanism by which lipid imbalance turns out to be toxic is lipid peroxidation. Lipid peroxides are highly reactive compounds, which trigger many pathological states including inflammation, cancer, neurodegenerative disease, as well as ocular and kidney degeneration. They result from enzymatic (cyclooxygenase (COX), cytochrome P450 (CYP), and lipoxygenase (LOX)) and non-enzymatic (“Fenton Chemistry”) peroxidation reactions of lipids, preferentially PUFAs. Lipid peroxides not only alter the assembly, composition, structure, and dynamics of cell membranes, but can exhibit additional toxicity with their degradation products: hydroxy acids and reactive aldehydes, like and 4-hydroxynonenal (4-HNE) and MDA, are able to propagate further generation of reactive oxygen species (ROS) or crosslink DNA and proteins, resulting in multiple pathologies and cell death [74,75,76,77].

To conclude, Fas bioavailability, bioaccessibility and toxicity contribute to determine their role as regulatory and bioactive molecules.

## 4. Crosstalk between Fatty Acids and Inflammation

FAs, synthesized or metabolized from dietary sources, serve mainly as structural components of cells and energy substrates; however, increasing evidence have demonstrated an important role as mediators of inflammation, with downstream effects depending on their nature [78,79]. The effect of FAs on inflammatory responses derives from their incorporation into cell membrane phospholipids. Different lipid mediators may be generated from phospholipids, which can boost or fight against inflammation. Based on that, lipid mediators deriving from ω-6 PUFAs are considered pro-inflammatory and are associated with the pathogenesis of inflammatory processes [80]. A direct link with inflammation is provided by LA, a common dietary ω-6 PUFAs. LA can be converted to ARA, a major component of membranes phospholipids, which acts as a substrate for COX, LOX, and CYP enzymes to yield eicosanoid mediators, such as hydroperoxyeicosatetraenoic acid (HPETE), prostaglandins (PGs), thromboxanes (TXs), leukotrienes (LTs), and lipoxins. Given its abundance in cell membranes, ARA is the major substrate for eicosanoids production, which explains why it is considered proinflammatory. Eicosanoids are oxidized lipid derivatives, which control initiation, magnitude, and duration of inflammation, acting usually via G protein-coupled receptors (GPCRs) [81]. However, not all eicosanoids have pro-inflammatory effects, such as those derived from ω-3 PUFAs [82]. In this regard, EPA and DHA, resulting from chemical modifications of ALA, the major representative of ω-3 PUFAs, give rise to eicosanoids with primarily anti-inflammatory or pro-resolving functions [82]. Because of structural differences compared to those derived from ARA, EPA-derived eicosanoids bind with lower affinity to eicosanoid receptors resulting in a weaker inflammatory response [83]. Moreover, EPA and DHA give rise to pro-resolving eicosanoid mediators, the so-called resolvins, protectins, and maresins, which accelerate the resolution of inflammation [84,85], inhibiting immune cells migration, matrix metalloproteinases synthesis, and the release of inflammatory cytokines, like interleukine (IL)-1α, IL-1β, IL-6, IL-8, IL-12, tumor necrosis factor α (TNFα), interferon γ (IFNγ), and many more [83]. The main mechanism by which ω-3 PUFAs block the cytokines storm is through the suppression of the nuclear factor kappa B (NF-κB) inflammatory signaling [86] NF-κB impairment is firstly mediated by the activation of the peroxisome proliferator-activated receptor γ (PPAR-γ), a transcription factor that physically interferes with NF-κB translocation to the nucleus, preventing pro-inflammatory cytokines release [87]. Moreover, ω-3 PUFAs prevent the activation of Toll-like receptors (TLRs), in particular TLR4, which is the starting point for NF-κB activation and, lastly, they initiate an anti-inflammatory signaling cascade by binding to GPR40 and GPR120 [88,89,90]. The different effects of dietary ω-6 and ω-3 PUFAs on the inflammatory cascade shed light on the importance of keeping a low ω-6/ω-3 ratio (4:1), in order to prevent or mitigate inflammatory-based pathologies, such as obesity, T2DM, arthritis, and others [91,92,93]. 

Anti-inflammatory properties have also been attributed to MUFAs [94]. MUFAs, such as oleic acid or palmitoleate, inhibit the formation of nucleotide-binding and oligomerization domain-like receptor, leucine-rich repeat, and pyrin domain-containing 3 (NLRP3) inflammasome, which is important for the production of mature inflammatory cytokines (i.e., IL-1β and IL-18) [7]; moreover, they maintain 5’AMP-activated protein kinase (AMPK) activity, which reduces the inflammatory response [95]. As PUFAs, MUFAs do not seem to activate TLR4 signaling [96], which instead, is one of the main mechanisms by which SFAs promote inflammation. Indeed, a proinflammatory effect has been attributed to SFAs, especially lauric acid, because of their effect on TLR2 and TLR4 signaling pathways [96,97]. SFAs trigger the dimerization and translocation of TLR2 or TLR4 into lipid rafts of plasma membranes, activating downstream signal transduction pathways in monocytes/macrophages, adipocytes, skeletal muscle cells, and pancreatic β cells [97,98]. Upon interaction with TLRs, SFAs activate the myeloid differentiation primary response 88 (MyD88)-dependent signaling cascade, culminating with the activation of the two major inflammation-related signaling routes in cells, NF-κB and mitogen-activated protein kinase (MAPK) pathways [97]. Both result in increased expression and release of pro-inflammatory cytokines and chemokines. These mediators induce local or systemic inflammation, also helped by the massive release of ROS upon activation of nicotinamide adenine dinucleotide phosphate (NADPH) oxidase [99]. The signaling cascade activated by ROS and other stress molecules, like ATP and ceramides, leads to the assembly of the NLRP3 inflammasome, with the subsequent release of inflammatory cytokines [100,101], which, on the contrary, is counteracted by dietary MUFAs and ω-3 PUFAs [7,102]. In addition, SFAs may also induce the release of inflammatory cytokines in a MyD88-independent manner, via the activation and nuclear translocation of interferon regulatory factor 3 (IRF3), or reinforcing NF-κB activation [97].

However, SFAs must not be demonized in toto. A subset of SFAs, the so-called short chain fatty acids (SCFAs) are known for their positive regulatory effect on the immune responses. SCFAs are the end products of anaerobic fermentation of dietary fiber and resistant starches by colonic bacteria strains, with the main members being acetate, propionate, and butyrate. Recent evidence shows that SCFAs play an anti-inflammatory role through GPCRs, in particular GPR41 and GPR43, and via inhibition of histone deacetylases (HDACs) [103]. The binding of SCFAs to GPCRs prevents NF-κB activation [104] and up-regulates the expression of anti-inflammatory IL-10 via HDAC inhibition [105,106,107]. Although SCFAs anti-inflammatory role appears evident, some studies have found that the same pathway mediated by GPCRs may also trigger SCFAs-induced pro-inflammatory responses [108,109].

Moreover, other experimental studies have demonstrated that the effect of lipids can be modulated by other dietary components. For example, a diet containing SFAs supplemented with polyphenols (i.e., epigallocatechin gallate, resveratrol) shows a reduced pro-inflammatory response [110,111,112].

Remarkably, it has been found that the deficiency of NLRP3 inflammasome reduces the negative impact of a high fat diet (HFD) also through changes in the gut microbiota composition, supporting a pivotal role of this pathway in regulating the effect of fatty acids on inflammation also through the microbiome (see Section 6 for details) [113].

In conclusion, FAs exposure and FAs composition of cellular membranes profoundly influence cellular functions, but the final effect is complex and is the resultant of the whole composition of the diet. Overall, the types of FAs consumed with diet are of pivotal importance for the modulation of inflammatory processes via the activation or repression of key intracellular signaling pathways, with MUFAs, ω-3 PUFAs, and SCFAs mainly triggering anti-inflammatory responses, and SFAs or ω-6 PUFAs leading towards a pro-inflammatory state (Table 1). 

## 5. Nutrigenomics of Fats: Evidence from Animal Studies

Experimental studies in animal models, conducted under ethical conditions, have provided the opportunity to evaluate molecular effects of various lipid mixtures or individual molecules, which could not be otherwise obtained from human studies. Indeed, animals fed with SFAs, MUFAs, or PUFAs showed differences in the activation of inflammatory pathways in healthy and unhealthy conditions, as well as contributed to elucidate the molecular mechanisms by which dietary lipids can modulate these metabolic pathways through gene expression changes. 

For example, obese mice fed with a HFD (29.64% SFA, 4.86% PUFA) for 25 weeks developed a severe early psoriasiform skin inflammation, which was dependent on FFAs levels but independent of fat mass extension, blood glucose levels, and adipose tissue-derived mediators. SFAs, which were increased by the HFD, were unraveled to sensitize myeloid cells, resulting in an amplified proinflammatory immune response to TLRs stimuli, which in turn augmented keratinocyte activation. On the other hand, dietary reduction of SFAs (0.73% SFA, 3.73% PUFA) dampened psoriasiform inflammation [114]. In another animal model, non-obese non-diabetic mice gavaged for one day with PO (2 g/kg body weight), included in a low-fat diet (LFD) (13% of calories derived from fat, 17 kJ/g), showed an increase in hepatocellular lipids concentrations, lipid oxidation, and insulin resistance. Moreover, mouse transcriptomics revealed that PO differentially regulates pathways, including members of the TLRs and PPAR families, and NF-κB and TNF-related weak inducers of apoptosis [115]. Other experimental animal studies suggested that dietary SFAs (99.8% fat for 18 weeks) may directly promote the development of heart failure by inducing lipotoxicity [116]. SFAs, like lauric acid (C12:0) and myristic acid (C14:0), induced oxidative stress and myocardial fibrosis even in the absence of pressure overload if supplemented to a standard chow diet supplemented with 0.2% cholesterol and 10% CO diet for eight weeks *ad libitum* in female C57BL/6 mice. This supplementation enhanced myocardial fibrosis, reduced capillary density, and increased myocardial cell apoptosis in postoperative pathological myocardial hypertrophy. Overall, it aggravated the pressure overload-induced cardiomyopathy [117]. High intake of SFAs, especially in presence of carbohydrates, has been shown to increase blood cholesterol, TG, and low-density lipoprotein cholesterol (LDL-C), which are associated to arterial lumen stenosis, atherosclerosis development, and increased risk of coronary heart disease. Furthermore, animal studies also correlated HFD (60% pork lard for 16 weeks) to cognitive impairments [118]. 

Interestingly, the nutrigenomic effects of SFAs have been investigated and strongly differed from those of MUFAs. SFA-HFD containing 45% kcal palmitic acid and MUFA-HFD containing 45% kcal OA were fed to the eight to nine-week-old male C57BL/6 mice for 24 weeks. Despite an increased prevalence of obesity being observed in both HFD-contained groups, adipose IL-1β inflammation and insulin resistance were significantly lower in MUFA-HFD mice compared with the SFA-HFD group. A different regulation of IL-1β production was observed; SFA-HFD induced pro-IL-1β and NLRP3-mediated activation through the down-regulation of AMPK. On the contrary, MUFA-HFD suppressed IL-1β activation via AMPK-mediated NLRP3 [7]. Compared with SFA-HFD, this bi-directional regulation of AMPK and IL-1β was mainly identified in the adipose tissue, where MUFA-HFD retained adipose AMPK and weakened the activation of IL-1β. In another study, LFD and SFA-HFD (45% kcal PO) were used to feed seven to nine-week-old male C57BL/6J mice for 32 weeks consecutively. At the same time, another group of mice was fed with SFA-HFD for 16 weeks and then switched to MUFA-HFD (45% kcal sunflower oil) for another 16 weeks (SFA-to-MUFA-HFD). In SFA-HFD group, the expression of genes involved in β-cell differentiation, proliferation, and identity (such as *Ins2, Nkx6.1, Ngn3, and Rfx6,* as well as *Pdx1* and *Pax6*) was significantly reduced in pancreatic islet; this SFA-HFD-induced down-regulation was related to the impairment of cell functions. Interestingly, SFA-to-MUFA-HFD reduced hyper-insulinemia, pancreatic inflammation, and the progression of β-cell function impairment induced by SFA-HFD, which may mediate its effects in an IL-1β-AMPK-dependent manner [119]. This outcome highlights how the substitution of SFAs with MUFAs can restore impaired cellular responses. Indeed, other animal studies also showed that the detrimental effects of an unbalanced intake of dietary lipids can be reversed. In a study protocol, 12 week-old male C57BL/6 J mice were fed either a LFD or HFD (containing either 10 or 60% of the total caloric intake from fat, for 3 days, 1 week, or 2 weeks); one group of mice was fed the HFD for one week and then returned to the LFD for a further week. Results showed that after three days, one week, and two weeks, the HFD induced changes in the hippocampal proteins involved in metabolism, inflammation, cell stress, cell signaling, and cytoskeleton. However, the replacement of the HFD after one week by a LFD for a further week resulted in a partial recovery of the hippocampal proteome [120]. 

Concerning PUFAs, recent findings have shown that ω-3 and derivatives (0.31% or 1.25% of DHA for 4–5 weeks) can reduce the stimulation of inflammatory factors and promote the regression of inflammation during cardiovascular processes [121]. Nine-week-old male C57BL/6 ApoE^−/−^ mice were fed a HFD containing 20% lard for four weeks, and then fed with both 1% dietary DHA incorporated into phospholipids (DHA-PL) and EPA incorporated into phospholipids (EPA-PL) for another eight weeks. The results showed that DHA-PL and EPA-PL could markedly reduce the atherosclerotic lesions in ApoE^−/−^ mice. EPA-PL significantly reduced serum and liver lipid levels by mediating mRNA and protein levels of genes involved in hepatobiliary sterol metabolism, while the transcription of TNF-α, IL-6, and IL-1β was decreased by DHA-PL treatment [122]. The DHA-PL exerted mainly anti-inflammatory functions, while the EPA-PL was able to improve lipid metabolism in atherosclerosis. To study whether the anti-atherosclerotic effect of EPA-rich fish oil partly depends on the chemical group at ω-3 position, the 18–19 week-old male ApoE^−/−^ mice (C57BL/6J background) were used and clustered into three groups. In the model group, animals were fed with a HFD, while in the two study groups, animals were fed with a HFD containing 1% EPA-PL or 1.1% EPA incorporated into triglycerides (EPA-TG) (providing equal amounts of EPA) for 8 weeks. Compared with the control group, both EPA-PL and EPA-TG showed a reduction in the aortic atherosclerotic lesion area and inhibition of inflammatory markers in the aorta and circulation, with a better effect in presence of EPA-PL than EPA-TG. It should be noted that the serum and liver lipid levels of EPA-PL were lower than those of the model group, while EPA-TG only reduced the liver TG level. It has been proposed that EPA-PL has some initial advantages over EPA-TG in terms of bioavailability and therapeutic efficacy, which may be due to their structural differences at the ω-3 site [123]. In addition, ω-3 PUFAs can reduce blood pressure in rats, and the effect may depend on the membrane of the ion channels [124,125]. Due to positive effects on vascular function and blood pressure, but also due to their nutrigenomic properties, ω-3 PUFAs (EPA 3.7 g/kg/day and DHA 3 g/kg/day for 20 weeks) are considered promising anti-atherosclerotic agents [126,127].

In addition, ω-3 PUFAs can play an important role in central metabolism. Intracerebroventricular infusion of resolvin D1 (10 ng) and resolvin D2 (10 ng), bioactive lipid mediators generated from the ω-3 PUFAs DHA and EPA, produced antidepressant effects in male BALB/c mice (8–12 weeks old) via the mammalian target of rapamycin complex 1 signaling pathway, at the medial prefrontal cortex and dentate gyrus, which are important brain regions for these antidepressant effects [128]. Wen et al., studied in an animal model of Alzheimer’s disease (AD), obtained treating male Sprague Dawley rats with Aβ_1-40_, the protective effect of low (150 mg/kg body weight) and high (300 mg/kg body weight) doses of EPA-PL for 27 days. The results showed that both low and high dose of EPA-PL improved cognitive performance in Morris Water Maze in AD rats [129]. Furthermore, treatment with EPA-PL increased the time of target phase and the numbers of crossing platform. Meanwhile, EPA-PL groups also significantly decreased the level of MDA, apoptosis, hyper-phosphorylated Tau, CD11b, glial fibrillary acidic protein (GFAP), IL-1β, TNF-α, and increased the level of SOD in the brain of Aβ_1-40_-induced rats [129]. 

Finally, effects of ω-3 PUFAs on DNA methylation in tumors have also been observed [130,131]. Colorectal cancer (CRC) rat model was developed using N-methyl phosphite nitrourea, and six-week-old rats were fed with ω-3 PUFAs (1 g/kg body weight) for 12 weeks. The results showed that tumor incidence in ω-3 treated rats was much lower than in CRC model rats, suggesting a significant protective role of ω-3 PUFAs. Furthermore, 5-methylcytosine content in ω-3 PUFAs treated rats was higher than in CRC model rats, suggesting that ω-3 PUFAs might regulate cytosine methylation. Therefore, it has been hypothesized that ω-3 PUFAs could inhibit tumor growth through the regulation of DNA methylation [132]. Indeed, despite some contrasting findings [133,134], the involvement of epigenetic pathways in the mediation of the molecular effects of HF diets has been described. For example, methyl donor supplementation containing 5 g/kg diet of betaine, 5.37 g/kg of choline, 5.5 mg/kg of folic acid, and 0.5 mg/kg of vitamin B12 in HFD-fed for eight weeks *ad libitum* Wistar rats prevented the HFD-induced fat accumulation in the liver and modified mRNA hepatic profile, as well as the methylation of specific gene promoters [135]. Interestingly, it has been demonstrated that detrimental effects of HFD can be reversible, at the level of both the epigenotype and the phenotype [136].

In conclusion, animal studies highlight that dietary fatty acids not simply are a major energy source for the body, but also contribute to modulate various biological responses accordingly to their chemical features (Table 2). 

## 6. Nutrigenomics of Fats: Evidence from Human Studies

Growing evidence has shown that FAs may exert many of their biological effects by regulating the activity of several transcription factors [137]. For example, ω-3 FAs can influence the expression of several genes involved in lipid and carbohydrate metabolism, thermogenesis, and inflammatory processes by inducing sterol regulatory element binding proteins (SREBPs), PPARs, carbohydrate response element binding protein (ChREBP), and NF-κB [138]. 

As aforementioned, ω-3 PUFAs are natural PPARs ligands. In this context, a short-term DHA-rich fish oil supplementation (2400 mg/day for 8 weeks) increased PPARγ activity in peripheral blood mononuclear cells (PBMCs) of subjects with T2DM compared to placebo, improving lipid metabolism and energy homeostasis [139]. In agreement with such trial, the intake of ω-3 FAs from flaxseed oil (2 × 1000 mg/day, containing 400 mg α-linolenic acid, for 6 weeks) induced upregulation of *PPARγ* and low-density lipoprotein receptor (*LDLR*) genes and downregulation of *IL-1* and *TNF-α* in PBMCs of subjects with gestational diabetes compared to placebo. In addition, ω-3 supplementation also improved glycemic and lipid profiles, inflammatory markers, and oxidative stress when compared to placebo [140]. Moreover, another study demonstrated that supplementation with ω-3 PUFAs (2700 mg/day) enhanced nuclear factor erythroid 2-related factor 2 (*NRF2*) gene expression in PBMCs of subjects with T2DM [141]. NRF2 is a transcription factor that regulates the expression of antioxidant proteins that protect against oxidative damage triggered by injury and inflammation [142].

Regarding Parkinson disease, supplementation with 1000 mg/day of ω-3 FAs from flaxseed oil and 400 IU/day of vitamin E for 12 weeks significantly improved gene expression of *TNF-α*, *PPARγ* and *LDLR*, but did not affect *IL-1* and *IL-8* compared to placebo [143]. Additionally, supplementation with 50,000 IU of vitamin D every two weeks plus 2000 mg/day of ω-3 FAs from fish oil for 12 weeks also presented beneficial effects on gene expression of *IL-1* and vascular endothelial growth factor (*VEGF*) among women with polycystic ovary syndrome [144].

Genes involved in lipid metabolism were differentially expressed in PBMCs depending on ω-3 and ω-6 plasmatic levels and on the ratio of SFAs to PUFAs in healthy subjects [145]. In subcutaneous adipose tissue of subjects with obesity, high medium-chain-SFAs diet led to upregulation of genes related to citric acid cycle and oxidative phosphorylation, and downregulation of genes related to complement system and inflammation [146]. Moreover, the replacement of SFAs with PUFAs induced upregulations of liver X receptor-alpha (*LXRA*) and *LDLR*, while the expression of several LXRA target genes and genes involved in inflammation was downregulated in PBMCs of healthy subjects with moderate hypercholesterolemia [147]. Taking all this information together, diet rich in ω-3 may be beneficial for human health. However, further studies are necessary to elucidate the ω-3 effects in the pathogenesis of several diseases [138].

Mediterranean diet (MedDiet) has been proven to be highly effective in the prevention of cardiovascular diseases and cancer and in decreasing overall mortality. In this context, MedDiet, especially if supplemented with extra virgin olive oil (EVOO), can exert health benefits through inducing changes in gene expression in pathways associated with cardiovascular risk [148]. Additionally, MedDiet enriched in MUFAs with EVOO can module the postprandial antioxidant profile, and the expression of proinflammatory and oxidative stress-related genes, including NF-kB p65, monocyte chemoattractant protein-1 (*MCP-1*), *TNF-α*, NF-κB inhibitory α (*IkBα*), *SOD1*, upstream transcription factor 1 (*USF1*), and catalase [149,150]. The consumption of a functional VOO enriched with olive oil (250 mg/kg) and thyme phenolic compounds (250 mg/kg) upregulated the expression of key cholesterol efflux regulators (CYP27A1, CAV1, LXRB, RXRA) and nuclear receptor-related genes (PPARb/*γ*) [151]. 

Moreover, a study on 36 representative individuals selected within the PREvención con DIeta MEDiterránea (PREDIMED—Navarra) trial showed that nuts and EVOO were able to change the DNA methylation profile of several genes in peripheral blood leukocytes, with potentially beneficial effects for health (concerning genes related to intermediate metabolism, diabetes, inflammation, and signal transduction) [152]. This evidence contributes to explain the role of MedDiet and fat quality on health outcomes [153].

On the basis of gene-dietary fat interaction, several single nucleotide polymorphisms (SNPs) modulate the effects of certain dietary factors or food preferences, and the main results are shown in Table 3. Among these genes, the most studied ones are adiponectin, C1Q, and collagen domain containing (*ADIPOQ*), apolipoproteins, C-reactive protein (*CRP*), fatty acid desaturase 1 (*FADS1*), fatty acid desaturase 2 (*FADS2*). 

The *ADIPOQ* gene encodes for the adiponectin protein, which exerts anti-inflammatory, antioxidant, and insulin sensitizer effects [154]. Several SNPs in *ADIPOQ* gene have been associated with serum adiponectin levels (Table 3), interacting with dietary FAs [155,156,157]. Additionally, the rs2241766 SNP interrelates with ω-3 FAs changing serum adiponectin levels and other inflammatory biomarkers [155,156].

On the other hand, two SNP located at *APOE* gene (rs7412 and rs429358) modify apolipoprotein E mRNA codon 112 and 158, where cysteine is replaced by arginine in both positions, and hence forms three possible isoforms of the protein according to the resultant salt-bridges: ε2, ε3, and ε4 [158,159,160,161]. In this way, after eight weeks of a high-SFAs diet [162], increased CRP levels was observed in *APOE* ε4 carriers (high risk allele). In the same way, in the Food4Me study, the ε4 allele was associated with higher total cholesterol compared to non-risk genotypes [161]. Additionally, a 16-week dietary intervention with a replacement of 9.5% energy of MUFAs or ω-6 FAs, reduced plasmatic CRP levels in *APOE* ε4 carriers, while *APOE* ε3/ε3 carriers showed an opposite increment in CRP concentration [163]. 

Reports indicate that SNPs in *CRP* gene can change plasma CRP levels [164,165,166]. Additionally, higher intake of total PUFAs was associated with lower CRP levels, suggesting reduced chronic systemic inflammation [167]. Additionally, greater adherence to the MedDiet and particularly its fish component was associated with lower CRP blood levels, especially in those at highest genetic risk due to the *CRP* rs3093068 SNP [166]. Moreover, SNPs in *CRP* gene modulated the risk of inflammation, which depends on individual plasma fatty acid and lipid profile [168].

Recent data suggest that there is an association between fatty acid desaturase (*FADS1-3*) SNPs and lipid composition in human blood and tissues [169]. A systematic review identified that *FADS* SNPs may influence plasma and erythrocyte FA composition, as well as T2DM risk markers, such as HOMA-IR and fasting glucose [170]. In the same way, the presence of two common *FADS* haplotypes influences the ability to synthesize essential ω-3 and ω-6 LC-PUFAs (e.g., ARA and DHA). Additionally, the haplotype D was associated with increased FADS activity [171]. The *FADS1* rs174537 SNP impacts the synthesis of ARA and the overall capacity of whole blood to synthesize 5-lipoxygenase products [172]. Additionally, the rs174537T allele was associated with decrease in DGLA, ARA levels [173], and diminished delta-5 desaturase enzyme activity, and *FADS1* gene expression [174]. Another common *FADS1* SNP, rs174550, modifies metabolic responses to an LA-enriched diet (6% of total energy), influencing serum CRP and plasma fasting glucose levels and ARA proportion in men [175]. Considering that *FADS1* and *FADS2* code for key enzymes for the conversion of the ω-6 and ω-3 C18 FA to their respective C20 and C22 products, SNPs in these genes could alter the viability of these products.

In the context of nutrigenetics, it is difficult to predict the impact that a variant may have on the gene-diet interaction, especially because the genetic variation differs across populations. For example, the frequency of *FADS* polymorphisms changes according to the population, which could alter the efficiency of ω-6 and ω-3 in the diet [176]. The results of the aforementioned studies suggest that the interaction between genetic factors, especially SNPs, and dietary components can alter the response to nutritional interventions and, consequently, susceptibility to several diseases. 

Although we already know the gene-diet interaction, current strategies are based on nutritional recommendations for populations in general, not considering the influence of genetic factors on dietary components, such as lipids. Among the lipids-gene interaction mentioned in this section, most studies have demonstrated a crosstalk between ω-3 and genetic polymorphisms, as evidenced in Table 3. In this sense, a genetic risk score constructed from genes identified by genome association studies, partially explained the variation in changes in TG in response to supplementation with ω-3 FAs [5 g/day of fish oil (1.9–2.2 g EPA and 1.1 g DHA daily)] [177]. This finding, added to the others previously mentioned in this section, reinforces the importance of knowing the individual’s genetics background for the prescription of nutritional supplementation. 

In this context, precision nutrition is an important part of personalized medicine and an emerging approach to disease prevention and treatment that takes genetic information in consideration, as well as age, sex, pathophysiological status, and environmental issues, including dietary patterns [178,179]. Nutritional prescriptions should consider the diversity in the genetic background between individuals and ethnic groups, as it affects nutritional needs, metabolism, and the response to nutritional and dietary interventions. Thus, knowledge about nutrigenetics and nutrigenomics can allow health professionals to decide on the most appropriate treatment to achieve precision nutrition, as well as permitting the design of innovative strategies for the prevention, control, and treatment of several diseases [176,179].

Although nutrigenomics is extremely important for the implementation of precision nutrition, these studies still have limitations. First, several studies are still scarce and heterogeneous, which makes it difficult to compare apparently similar studies that analyze the same genetic variants and dietary components. Second, genomic data requires a comprehensive analysis of bioinformatics, which makes it difficult to understand the results. Third, it is necessary to integrate the results of several omics studies in order to have more robust findings. 

**Table 3:** Gene-dietary fat interactions mediating inflammation and lipid metabolism. ↑ higher levels; ↓ lower levels.

Gene	SNP	Dietary Fat Interaction	Main Results	References
*ADIPOQ*	rs2241766, rs16861209, rs17300539	**SFA** [C14:0 + C16:0 + EA], **MUFA** [C16:1n-7 + AO], **PUFA** [ω-6 PUFAs + ω-3 PUFAs], **ω-6** [LA (18:2 n-6) + DGLA (20:3 n-6) + AA (20:4n-6)], **ω-3-HUFA** [EPA + DPA (22:5 ω-3) + DHA], and **ω-3** [LA (18:3n3) + ω-3 HUFA].	rs2241766G allele: ↑ total plasma ω-3 FA content was protective against inflammation. Gene-plasma FA profile interaction: rs2241766 and ω-3; rs16861209 and ARA and DPA; rs17300539 and SFA.	[155]
	rs17300539, rs182052, rs16861209, rs1501299	**High-MUFA**: total fat 38%, carbohydrate 45% of energy. MUFA intake of 20% of energy.	rs182052 G/G genotype: serum adiponectin levels ↑ in 3.8% after a high-MUFA diet. In these patients, a high-MUFA diet may help to ↑ adiponectin concentrations with advancing age.	[157]
	rs17300539, rs2241766	**ω-3 PUFA** (fish oil supplementation—daily doses of 0.45, 0.9, and 1.8 g 20:5n3 and 22:6n3 (1.51:1), or placebo).	rs17300539A allele: ↑ serum adiponectin levels. rs2241766 T/T genotype: subjects aged >58y had a 22% ↑ in serum adiponectin levels compared to baseline after the highest dose of 20:5n3 and 22:6n3.	[156]
*APOE*	rs429358, rs7412	**SFA** (Food4Me Study)	*APOE* ε*4* allele was associated with higher total cholesterol.	[161]
	rs429358, rs7412	**Low-fat diet** (24% from fat, 8% from SFA, 59% from carbohydrate), **high-fat high-SFA diet** (38% from fat, 18% from SFA, 45% from carbohydrate), and **high-fat high-SFA diet supplemented with 3.45 g DHA/d**	*APOE* ε*4* carriers: ↑ CRP plasma levels after eight weeks of a high-SFA and high-SFA-DHA diets relative to low-fat diet.	[162]
	rs429358, rs7412	SFA with MUFA or ω-6FA	Diet-genotype interaction: differential responsiveness to MUFA intake between ε3/ε3 and ε*4* carriers.	[163]
*CRP*	rs2808630, rs3093058, rs3093062	SFA and MUFA	Presence of rs3093058 and rs3093062 minor allele: ↑ CRP levels in the presence of ↑ triglyceride or cholesterol intake. rs2808630 minor allele: ↑ intake of SFA and MUFA, ↑ CRP levels. Presence of the minor allele of these 3 SNPs: ↑ ω-6 to -3 ratio	[165]
	rs1205, rs1417938, rs2808630	FA	*CRP* SNPs modulated the risk of being in the inflammatory group depending on individual plasma FA and lipid profile.	[168]
	rs3093068, rs1130864, rs1205	MedDiet	The minor allele of rs3093068 and rs1130864: ↑ CRP levels rs1205T allele: ↓ CRP concentrations. Interaction between rs3093068 and MedDiet.	[166]
*FADS* cluster	*FADS1*: rs174537; *FADS2*: rs174575, rs2727270; *FADS3*: rs1000778	ω-3 and ω-6	The presence of rs174537, rs174575, and rs2727270 minor alleles: ↑ LA levels rs174537T and rs2727270T: ↓ DGLA and ARA levels rs1000778T allele: ↓ ARA levels	[173]
FADS haplotype	28 closely linked SNPs	ω-3 and ω-6	Two common FADS haplotype differ in their ability to generate LC-PUFAs.	[171]
*FADS1*	rs174537	ARA/LA	rs174537 impacts the synthesis of ARA and the overall capacity of whole blood to synthesize 5-lipoxygenase products.	[172]
	rs174537	PUFA	rs174537T allele carriers: ↓ in 20:4 ω-6 levels, ↓ delta-5 desaturase enzyme activity, and ↓ *FADS1* gene expression.	[174]
	rs174550	Habitual diet with a supplement of 30, 40, or 50 mL (27–45 g) sunflower oil (62% of LA) daily depending on BMI	In men carrying the T/T genotype, plasma eicosanoid concentrations correlated with the ARA proportion and with hsCRP. No correlations were found for C/C genotype.	[175]

## 7. Dietary Lipids Modulate Gut Microbiota Composition and Metabolites Production

Microbial community in humans reaches the highest density and taxonomic diversity in the gastrointestinal tract, with an estimation of 10^14^ microorganisms and almost 2000 different species of bacteria in the colon [180]. Owing its multifaceted crosstalk with the host environment, the gut microbiota provides a range of beneficial properties to the host, with a significant impact on human health and disease [181]. Research about the gut microbiome has exponentially flourished in the last 20 years, providing also an insight into the dissection of the impact of different dietary patterns on the gut microbiota composition [182], including fats.

Despite most dietary FAs being absorbed in the small intestine, a certain percentage can reach the colon, directly modulating the gut microbiota composition and function [183]. Indeed, FAs have been found to have an antimicrobial activity [184] and also represent a substrate for bacteria [185], hence affecting gut metabolism and the host health.

Several animal studies investigated the impact of HFD enriched in SFAs (Table S1, Supplementary Materials): while bacterial diversity and richness did not show significant changes, in most of the studies SFAs intake induced a significant increase in the Firmicutes/Bacteroidetes ratio, which has been associated with obesity [186]. Negative effects of SFAs on the microbiota composition included the decrease in *Bifidobacterium*, a beneficial probiotics genus [187], the increase in *Clostridium*, a genus that also includes pathogenic strains [188], and the increase in *Bilophila wadsworthia*, a sulphide-producer bacteria, which has been associated with immune and metabolic impairments [189]. However, an increase in Ruminococcaceae, butyrate producing bacteria [190], has also been observed. Some studies also evidenced that a HFD leaded to gut-barrier alterations and consequent inflammation and gut-related disease [191,192,193].

Preliminary results from in vivo studies showed that a high intake of CC [194] or rapeseed oil [195], rich in medium-chain fatty acids (MCFAs), has a positive impact on gut microbiota in comparison to a diet rich in long-chain FAs, suggesting that the SFAs’ chemical structure (e.g., in the length of FAs chains) may differentially affect the microbiota composition [195].

Since it is well known that trans-FAs, originated from hydrogenated vegetable oils, contribute to the development of chronic diseases [196], two recent studies investigated their impact also on animals’ gut microbiota (Table S2, Supplementary Materials). They demonstrated that trans-FAs induce adverse effects, decreasing Bacteroidetes and increasing Firmicutes and Proteobacteria abundance [197,198], the latter considered a signature of dysbiosis [199]; also, the families Ruminococcaceae, Lachnospiraceae, and Rikenellaceae were significantly reduced in mice that consumed hydrogenated soybean oil [197], supporting the hypothesis of a negative impact of trans-FAs on the gut microbiota composition and on health. 

A few studies investigated the effect of MUFAs on the gut microbiota composition in animal models (Table S3, Supplementary Materials), suggesting an opposite effect than SFAs. In particular, MUFAs were associated with an increase in Bacteroidetes and a decrease in Firmicutes. 

More data are available on PUFAs’ effect (both ω-3 and ω-6, respectively described in Tables S4 and S5 Supplementary Materials). Overall, the regular consumption of ω-3 has been associated with a condition of eubiosis, increasing probiotic bacteria such as *Bifidobacterium, Lactobacillus*, and *Akkermansia* [200,201,202,203] and SCFAs-producer families, such as Ruminococcaceae and Lachnospiraceae [200]. On the contrary, in vivo studies, where ω-6 PUFAs were supplemented, showed a decrease in *Bifidobacterium* and *Lactobacillus* [192,204] and an increase in *Clostridium* [202,204], Firmicutes, and Proteobacteria [193]. 

Regarding studies on dietary lipids intake and gut microbiota in humans, a recent systematic review [205] analyzed and compared observational and interventional studies. Although the latter did not provide evidence of correlation between dietary fat intake and gut microbiota composition, observational studies evidenced interesting metabolic associations. Both types of studies showed a significant decrease in bacterial diversity and richness in participants consuming HFD enriched in SFAs. Differentially, a MUFAs-enriched diet reduced the number of total bacteria and PUFAs-enriched diet did not induce any significant change in richness, diversity, and total number of bacteria [205]. Results from observational studies demonstrated positive correlations between (i) SFAs intake and bacterial genera such as *Blautia* and *Clostridium*, (ii) MUFAs intake and *Blautia*, (iii) PUFAs intake and Tenericutes, the latter associated with lower TG in plasma. The higher ratio Firmicutes/Bacteroidetes in subjects consuming SFAs is in accordance with results from studies in mice and correlated with obesity and inflammatory conditions. On the contrary, this effect was not found in participants consuming diets enriched in MUFAs or PUFAs. For an in depth description, see [205,206,207]. 

Although most studies have been conducted in animals and results are still not consistent, interesting findings are revealing, suggesting that HFD rich in SFAs exerts negative effects on the gut microbiota with consequences on human metabolism and health [208]; a diet rich in PUFAs may have a positive effect on microbial communities in human gut, supporting the beneficial role of these essential and anti-inflammatory dietary components on human health. On the contrary, effects of a diet rich in MUFAs on the gut microbiome composition are still inconclusive. Further studies might support the promising, but still complex, design of personalized diets able to modulate the microbiota composition also through the effect of specific FAs.

## 8. Conclusions

**Figure 3:**
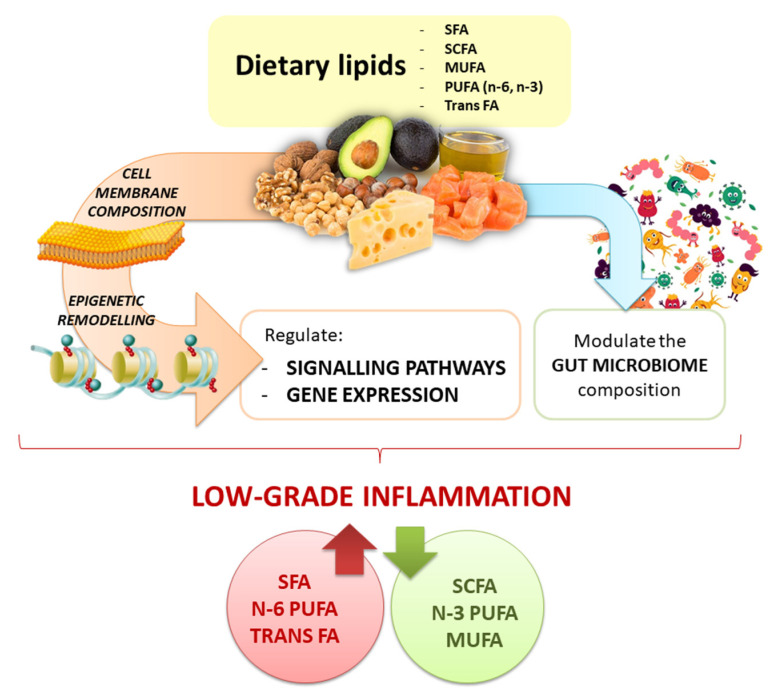
Graphical overview of the nutrigenomic impact of dietary lipids.

Plant- and animal-derived food differ in dietary lipid content and quality associated chemical characteristics, as well as for the presence of additional bioactive compounds and/or nutrients that contribute to the final effect on gene expression regulation (i.e., the nutrigenomic effect) (Figure 3). The chemical structure of lipids influences also their oxidative stability contributing to produce secondary metabolites with various impact on molecular responses. Research on animal models highlights how the type of FAs, their amount, and the time of exposure to a fat-rich diet can modulate cellular responses mediated by regulating gene expression, and significantly contributing to describe the nutrigenomic role of dietary lipids. However, going toward human studies, the picture becomes more complex, also because of the contribution of the individual genetic variability and the gut microbiota diversity. According to the gene-dietary fat interaction, SNPs contribute to produce multifaceted molecular responses, that may differ from outcomes obtained in vitro and/or in preclinical studies. Results from animal and human studies on gut microbiota composition partially contribute to characterize the response associated to dietary fat intake. 

A major strength of the research production in this area relies on the specific contribution to describing the complex impact of dietary lipids on gene expression regulation. However, a major weakness in the characterization of this multifaceted picture still exists, especially in humans. This is at least partially due to the wide extent of the human genetic variability, which generates a plethora of different phenotypes, with heterogeneous responses to dietary factors, including lipids. The incorporation of nutrigenetic data into human studies might help to reduce this confounding factor. Genomics might be used for Mendelian randomization studies aimed to demonstrate causal effects of lipids on human health and diseases, reducing confounding and reverse causation [209]. Finally, a better understanding of the role of the microbiome and of metabolites that it produces (still only partially characterized because of the complexity of the system and the large inter-individual variations) might help to elucidate how it modulates the effect of dietary lipids in each individual. 

In conclusion, to properly define the exact individual response to dietary lipids, the research on the nutrigenomics of dietary lipids urgently needs the confluence of omics domains (i.e., nutrigenomics, nutri-epigenetics, nutrigenetics, metabolomics, especially lipidomics, etc.) [210], which will strongly improve the possibility to translate this knowledge into clinical practice. 

## Figures and Tables

**Table 1:** Major molecular mechanisms explaining the dietary FAs modulation of the inflammatory response.

Fatty Acids	Pro-Inflammatory Effect	Anti-Inflammatory Effect	Other Effects	References
SFAs	TLR2/TLR4 signaling pathways			[96,97,98]
	MyD88-dependent NF-κB and MAPK activation			[97]
	IL-1α, IL-1β, IL-6, IL-8, IL-12, TNFα, IFNγ release			
	MyD88-independent IRF3, and NF-κB activation			
	NADPH oxidase activation and ROS release			[99]
	NLRP3 inflammasome assembly and activation			[100,101]
		Reduced pro-inflammatory response in combination with polyphenols (i.e., epigallocatechin gallate, resveratrol)		[110,111,112]
SCFAs		GPR41/GPR43-mediated signaling pathways		[103]
		Inhibition of NF-κB activation		[104]
		Anti-inflammatory IL-10 release via HDACs inhibition		[105,106,107]
	GPCRs-mediated inflammatory responses			[108,109]
MUFAs		Downregulation of IL-1β and IL-18 expression via NLRP3 inflammasome inhibition		[7]
		AMPK-mediated anti-inflammatory response		[95]
		Do not activate TLR2/TLR4 signaling pathways		[96]
ω-6 PUFAs	ARA-derived eicosanoids, such as HPETE, PG, TX, LT, and lipoxins, induce inflammatory response via GPCRs			[81]
			Promote obesity, T2DM, arthritis	[91,92,93]
ω-3 UFAs		EPA/DHA-derived eicosanoids, such as resolvins, protectins, and maresins, induce a milder inflammatory response and accelerate resolution of inflammation		[82,83,84,85]
		NF-κB signaling suppression via PPAR-γ activation, impairment of TLRs activation, and GPR40 and GPR120-mediated anti-inflammatory cascade		[86,87,88,89,90]
		NLRP3 inflammasome inhibition		[102]
			Contrast obesity, type 2 diabetes, arthritis	[91,92,93]

**Table 2:** Impact of dietary fatty acids on gene mediated inflammatory responses/outcomes: studies in animal models.

Fatty Acids	Pro-Inflammatory Effect	Anti-Inflammatory Effect	Other Effects	References
PO (2 g/kg body weight)			increase LOX and insulin resistance	[115]
HFD (29.64% SFAs, and 4.86% PUFAs)	IL-1β, IL-6, TNF-α, OP, Cox2, SA8, SA9, CXCL1, CCL3			[114]
SFAs (99.8% fat)			induce cardiac hypertrophy, left ventricular systolic, and diastolic dysfunction, and autophagy	[116]
SFAs (0.2% cholesterol and 10% CO)			reduce oxidative stress and myocardial fibrosis	[117]
HFD (60% pork lard)	IL-6, TNF-α, MCP-1		disrupt cognition	[118]
HFD (60% kcal)			metabolism, cellular stress responses, cyto-skeletal organization, cell signaling, and the immune system	[120]
MUFA-HFD (45% kcal (OA))		NLRP3, IL-1β, IL-18	improve insulin sensitivity	[7]
MUFA-HFD (45% kcal sunflower oil)		IL-1β, IL-6	attenuate hyperinsulinemia	[119]
PUFAs (0.31% or 1.25% of DHA)			reduce heart rate and arrhythmia vulnerability	[121]
DHA-PL, EPA-PL (1% dietary DHA or EPA incorporated into phospholipids)		TNF-α, IL-6, IL-1β, CD68	reduce atherosclerotic lesions	[122]
EPA-PL (1% EPA-PL)		CRP, TNF-α, IL-6, MCP-1	regulate cholesterolmetabolism	[123]
resolvin D1 (10 ng) and D2 (10 ng)			antidepressant, activation of mTORC1 signaling	[128]
EPA-PL (150 or 300 mg/kg body weight)		CD11b, GFAP, IL-1β, TNF-α	alleviate oxidative stress, and hyper-phosphorylated tau	[129]
ω-3 PUFAs (12% fish iol)			mtDNA methylation	[130]
ω-3 PUFAs (0.5% EPA and DHA)			DNA methylation	[131]
ω-3 PUFAs (1 g/kg body weight)			DNA methylation	[132]

## Supplementary Materials

The following are available online at https://www.mdpi.com/article/10.3390/antiox10070994/s1, Table S1: The impact of HFD rich in saturated fats on gut microbiota composition in animals, Table S2: The impact of TFA (trans fatty acids) on gut microbiota composition in animals, Table S3: The impact of MUFA (monounsaturated fatty acids) on gut microbiota composition in animals, Table S4: The impact of PUFA-n-3 (polyunsaturated fatty acids-omega3) on gut microbiota composition in animals, Table S5: The impact of PUFA-n-6 (polyunsaturated fatty acids-omega6) on gut microbiota composition in animals.

## Abbreviations

AD	Alzheimer’s disease
ADIPOQ	Adiponectin, C1Q and collagen domain containing
ALA	α-Linolenic acid
AMPK	5′AMP-activated protein kinase
ARA	Arachidonic acid
CO	Coconut oil
ChREBP	Carbohydrate response element binding protein
COX	Cyclooxygenase
CRC	Colorectal cancer
CRP	C-reactive protein
DHA	Docosahexaenoic acid
DGLA	Dihomo gamma-linolenic acid
EPA	Eicosapentaenoic acid
EA	Estearic acid
EVOO	Extra virgin olive oil
FAs	Fatty acids
FADS	Fatty acid desaturase
FFAs	Free fatty acids
GFAP	Glial fibrillary acidic protein
GPCRs/GPRz	G protein-coupled receptors
HDACs	Histone deacetylases
HFD	High-fat diet
HPETE	Hydroperoxyeicosatetraenoic acid
HUFA	Highly unsaturated fatty acid
IFNγ	Interferon γ
IL	Interleukine
IRF3	Interferon regulatory factor 3
LA	Linoleic acid
LDLR	Low density lipoprotein receptor
LFD	Low-fat diet
LOX	Lipoxygenase
LTs	Leukotrienes
LXRA	Liver X receptor-alpha
MAPK	Mitogen-activated protein kinase
MCP-1	Monocyte chemoattractant protein-1
MCFAs	Medium chain fatty acids
MedDiet	Mediterranean diet
MUFAs	Monounsaturated fatty acids
MyD88	Myeloid differentiation primary response 88
NADPH	Nicotinamide adenine dinucleotide phosphate
NAFLD	Non-alcoholic fatty liver disease
NF-κB	Nuclear factor kappa B
NLRP3	Nucleotide-binding and oligomerization domain–like receptor, leucine-rich repeat and pyrin domain–containing 3 inflammasome
NRF2	Nuclear factor erythroid 2-related factor 2
OA	Oleic acid
PBMCs	Peripheral blood mononuclear cells
PGs	Prostaglandins
PL	Phospholipids
PO	Palm oil
PPAR-γ	Peroxisome proliferator-activated receptor γ
PUFAs	Polyunsaturated fatty acids
ROS	Reactive oxygen species
SCFAs	Short chain fatty acids
SCI	Systemic chronic inflammation
SFAs	Saturated fatty acids
SNPs	Single nucleotide polymorphisms
SOD	Superoxide dismutase
SREBPs	Sterol regulatory element binding proteins
T2DM	Type 2 diabetes mellitus
TG	Triglycerides
TLRs	Toll-like receptors
TNFα	Tumor necrosis factor α
TXs	Thromboxanes
ω-3	Omega-3 fatty acids
ω-6	Omega-6 fatty acids
USF1	Upstream transcription factor 1
UCP2	Uncoupling protein 2
VEGF	Vascular endothelial growth factor
