# Supplementary material for: Nutrigenomics of Dietary Lipids
Source: Antioxidants (Basel). 2021 Jun 22;10(7):994. doi: 10.3390/antiox10070994 (PMC8300813; doi:10.3390/antiox10070994)
Supplement: Supplementary file 1 [file antioxidants-10-00994-s001.zip › antioxidants-1236011-supplementary.pdf]

**Table S1: The impact of HFD rich in saturated fats on gut microbiota composition in animals. Most significant and relevant changes are reported.**

| Ref. | Model | Lipid source | α-Diversity | Phylum | | | | Family | | | | | | | | Genus | | | | | |
|---|---|---|---|---|---|---|---|---|---|---|---|---|---|---|---|---|---|---|---|---|---|
| | | | | Firmicutes | Bacteroidetes | Proteobacteria | Actinobacteria | Ruminococcaceae | Lachnospiraceae | Rikenellaceae | Desulfovibrionaceae | Bacteroidaceae | Erysipelotrichaceae | Provotellaceae | Enterobacteriaceae | *Bifidobacterium spp.* | *Lactobacillus spp.* | *Allobaculum* | *Clostridium spp.* | *Bilophila* | *Turicibacter* |
| 1 | mice | Palm oil | / | ↑ | ↓ | ↑ | ↓ | | ↑ | | | ↓ | ↑ | | | | | | | | |
| 2 | mice | Lard | / | | | | | | | | ↑ | | | | | ↓ | | | | ↑ | |
| 3 | mice | SFA | / | | | | | | ↑ | | | | ↑ | | | ↓ | ↑ | ↓ | | ↑ | |
| 4 | mice | Palm oil | ↓ | ↑ | ↓ | | | | | | | | | | | | | | ↑ | | |
| 5 | mice | Milk fats | / | ↑ | ↓ | ↑ | | | | | | | | | | | | | | | |
| 6 | mice | HFD | / | | | | | | | | | | | | ↓ | ↓ | | | ↑ | | |
| 7 | mice | butter | ↓ | | ↓ | | | | | | ↑ | | | | | | ↑ | | | | |
| 8 | mice | Lard | ↑ | | | | | ↑ | ↓ | ↓ | ↑ | | | | | | | | | | |
| 8 | mice | Palm oil | ↑ | | | | | ↑ | ↑ | ↓ | ↑ | | | | | | | | | | |
| 9 | mice | HFD | / | ↑ | ↓ | ↓ | | ↑ | | ↑ | | ↓ | | ↓ | ↑ | ↓ | | | | | |
| 10 | mice | HFD | ↓ | ↑ | ↓ | ↑ | | ↑ | ↑ | | | | | | | ↓ | | | | | |
| 11 | mice | HFD | / | | | | | | | | | ↓ | | | ↑ | ↓ | | ↓ | ↑ | | ↑ |

| | | | | | | | | | | | | | | | | | | | | | |
|---|---|---|---|---|---|---|---|---|---|---|---|---|---|---|---|---|---|---|---|---|---|
| 12 | hens | HFD | / | ↑ | ↓ | | | | | | | | ↑ | | | | | | | | |
| 13 | rats | HFD | ↓ | | | | | | | | | | | ↑ | | | | | | | |
| 14 | mice | Coconut oil | / | ↑ | ↓ | | | | | | | | | | | ↑ | ↑ | ↑ | ↑ | | |
| 15 | mice | Rapeseed oil | / | ↓ | ↑ | ↓ | | | ↓ | | | | | | | | ↓ | | | | |
| 16 | rats | Coconut oil | ↓ | ↓ | ↑ | | | | | | | | | | | ↑ | | ↑ | | | |

/ no significant difference or not analysed; ↑ increase; ↓ decrease

**Table S2: The impact of TFA (trans fatty acids) on gut microbiota composition in animals. Most significant and relevant changes are reported.**

| Ref. | Model | Lipid source | α-diversity | Phylum | | | | Family | | | | | | | Genus |
|---|---|---|---|---|---|---|---|---|---|---|---|---|---|---|---|
| | | | | Firmicutes | Bacteroidetes | Proteobacteria | Actinobacteria | Deferribacteres | Ruminococcaceae | Lachnospiraceae | Rikenellaceae | Bacteroidaceae | Desulfovibrionaceae | Lactobacillaceae | *Lactobacillus spp.* |
| 17 | mice | hydrogenated soybean oil | ↓ | | ↓ | ↑ | ↑ | ↓ | ↓ | ↓ | ↓ | ↓ | ↑ | ↑ | ↑ |
| 18 | rats | hydrogenated soybean oil | / | ↑ | ↓ | ↑ | | | | | | | | | |

/ no significant difference or not analysed; ↑ increase; ↓ decrease

**Table S3: The impact of MUFA (monounsaturated fatty acids) on gut microbiota composition in animals. Most significant and relevant changes are reported.**

| Ref. | Model | Lipid source | α-diversity | Phylum | | | | Family | | | | Genus | | |
|---|---|---|---|---|---|---|---|---|---|---|---|---|---|---|
| | | | | Firmicutes | Bacteroidetes | Proteobacteria | Actinobacteria | Ruminococcaceae | Lachnospiraceae | Erysipelotrichaceae | Provotellaceae | *Bifidobacterium spp.* | *Lactobacillus spp.* | *Clostridium spp.* |
| [1] | mice | Olive oil | / | ↑ | ↓ | ↓ | ↓ | | | ↑ | | | | |
| [7] | mice | Olive oil | ↓ | | | | | | | ↑ | ↑ | | | ↑ |
| [19] | rats | Olive oil | ↑ | ↑ | ↓ | | ↓ | ↑ | ↑ | | | | ↑ | |
| [20] | mice | Oleic acid compounds | / | ↓ | ↑ | | | | | | | ↑ | ↓ | |
| [4] | mice | Olive oil | / | ↓ | ↑ | | | | | | | | | ↓ |

/ no significant difference or not analysed; ↑ increase; ↓ decrease

**Table S4: The impact of PUFA-n-3 (polyunsaturated fatty acids-omega3) on gut microbiota composition in animals. Most significant and relevant changes are reported.**

| Ref. | Model | Lipid source | α-diversity | Phylum | | | | Family | | | | | | | | Genus | | | | | | | | |
|---|---|---|---|---|---|---|---|---|---|---|---|---|---|---|---|---|---|---|---|---|---|---|---|---|
| | | | | Firmicutes | Bacteroidetes | Proteobacteria | Actinobacteria | Deltaproteobact ria | Ruminococcace ae | Lachnospiracea e | Rikenellaceae | Bacteroidaceae | Enterobacteriace ae | BIfidobacteriace ae | Erysipelotrichac eae | *Bifidobacterium spp* | *Lactobacillus spp.* | *Allobaculum* | *Turicibacter* | *Clostridium spp.* | *Akkermansia* | *Bilophila* | *Helicoacter* | *Pseudomonas* |
| [21] | mice | n-3 | / | | | | | | | | | | | | | ↑ | ↑ | | | | | | | |
| [1] | mice | Flaxseed/fish oil | ↑ | | | ↓ | ↑ | | ↑ | ↑ | | ↓ | | ↑ | ↑ | ↑ | | ↑ | | | | | | |
| [22] | mice | Fish oil | / | ↓ | | | | | | | | | | | | | | | | | | | ↓ | ↓ |
| [23] | mice | Fish oil | ↓ | | ↓ | | ↑ | ↑ | | | | | | | | ↑ | ↑ | | | | ↑ | ↑ | ↑ | |
| [3] | mice | n-3 | / | | | | | | | | | | | | | ↑ | ↑ | ↑ | ↑ | ↑ | ↓ | ↓ | | |
| [24] | mice | n-3 | ↓ | ↓ | ↑ | | | | | | | | ↓ | | | ↑ | | | | | | | | |
| [20] | mice | EPA/DHA | / | ↑ | | | | | | | | | | | | ↑ | ↑ | | | | | | | |
| [25] | Rats | Low n-6/n-3 | ↑ | | | | ↓ | | | | | | | | | | | ↑ | | | | | | |

/ no significant difference or not analysed; ↑ increase; ↓ decrease

**Table S5: The impact of PUFA-n-6 (polyunsaturated fatty acids-omega6) on gut microbiota composition in animals. Most significant and relevant changes are reported.**

| Ref. | Model | Lipid source | α-diversity | Phylum | | | | Family | | | | | | | Genus | | | | | | | |
|---|---|---|---|---|---|---|---|---|---|---|---|---|---|---|---|---|---|---|---|---|---|---|
| | | | | Firmicutes | Bacteroidetes | Proteobacteria | Actinobacteria | Ruminococcaceae | Lachnospiraceae | Rikenellaceae | Bacteroidaceae | Erysipelotrichaceae | Provotellaceae | Enterobacteriaceae | *Bifidobacterium spp.* | *Lactobacillus spp.* | *Allobaculum* | *Turicibacter* | *Clostridium spp.* | *Akkermansia* | *Oscillibacter* | *Bilophila* |
| 26 | Weaning mice | Soy oil | / | | | | | | | | | | | | ↓ | ↓ | | | ↑ | | | |
| 14 | mice | Soy oil | / | | | | | | | | | | | | | ↑ | | | | ↑ | | |
| 21 | mice | n-6 | / | | | | | | | | | | | ↑ | | | | | ↑ | | | |
| 1 | mice | Safflower oil | / | | | | | ↑ | ↓ | ↓ | ↑ | | | | ↑ | | ↑ | | | | ↑ | |
| 3 | mice | n-6 | / | | | | | | ↑ | | | ↑ | | | ↓ | ↓ | ↓ | | | | ↑ | ↑ |
| 4 | mice | Safflower oil | / | | ↑ | | | | | | | | | | | | | | ↑ | | | |
| 5 | mice | Safflower oil | / | ↑ | ↓ | ↑ | ↑ | | | | | | | | | | | | | | | |
| 25 | rats | High n-6/n-3 | ↑ | | | ↓ | | | | | | | | | | | ↓ | | | | | |

/ no significant difference or not analysed; ↑ increase; ↓ decrease

**BIBLIOGRAPHY**


1. Patterson, E. *et al.* Impact of dietary fatty acids on metabolic activity and host intestinal microbiota composition in C57BL/6J mice. *Br J Nutr* **111**, 1905–1917 (2014).
2. Shen, W. *et al.* Intestinal and systemic inflammatory responses are positively associated with sulfidogenic bacteria abundance in high-fat-fed male C57BL/6J mice. *J Nutr* **144**, 1181–1187 (2014).
3. Lam, Y. Y. *et al.* Effects of dietary fat profile on gut permeability and microbiota and their relationships with metabolic changes in mice. *Obesity (Silver Spring)* **23**, 1429–1439 (2015).
4. de Wit, N. *et al.* Saturated fat stimulates obesity and hepatic steatosis and affects gut microbiota composition by an enhanced overflow of dietary fat to the distal intestine. *Am J Physiol Gastrointest Liver Physiol* **303**, G589-599 (2012).
5. Huang, E. Y. *et al.* Composition of dietary fat source shapes gut microbiota architecture and alters host inflammatory mediators in mouse adipose tissue. *JPEN J Parenter Enteral Nutr* **37**, 746–754 (2013).
6. Yamada, S. *et al.* Gut microbiota-mediated generation of saturated fatty acids elicits inflammation in the liver in murine high-fat diet-induced steatohepatitis. *BMC Gastroenterol* **17**, 136 (2017).
7. Prieto, I. *et al.* Influence of a diet enriched with virgin olive oil or butter on mouse gut microbiota and its correlation to physiological and biochemical parameters related to metabolic syndrome. *PLoS One* **13**, e0190368 (2018).
8. Just, S. *et al.* The gut microbiota drives the impact of bile acids and fat source in diet on mouse metabolism. *Microbiome* **6**, 134 (2018).
9. Kim, K.-A., Gu, W., Lee, I.-A., Joh, E.-H. & Kim, D.-H. High fat diet-induced gut microbiota exacerbates inflammation and obesity in mice via the TLR4 signaling pathway. *PLoS One* **7**, e47713 (2012).
10. Zhang, C. *et al.* Structural resilience of the gut microbiota in adult mice under high-fat dietary perturbations. *ISME J* **6**, 1848–1857 (2012).
11. Martinez-Guryn, K. *et al.* Small Intestine Microbiota Regulate Host Digestive and Absorptive Adaptive Responses to Dietary Lipids. *Cell Host Microbe* **23**, 458-469.e5 (2018).
12. Liu, C. *et al.* Alterations and structural resilience of the gut microbiota under dietary fat perturbations. *J Nutr Biochem* **61**, 91–100 (2018).
13. Gual-Grau, A., Guirro, M., Mayneris-Perxachs, J., Arola, L. & Boqué, N. Impact of different hypercaloric diets on obesity features in rats: a metagenomics and metabolomics integrative approach. *J Nutr Biochem* **71**, 122–131 (2019).
14. Patrone, V. *et al.* Differential effects of coconut versus soy oil on gut microbiota composition and predicted metabolic function in adult mice. *BMC Genomics* **19**, 808 (2018).
15. Zhou, S. *et al.* Effects of Medium- and Long-Chain Triacylglycerols on Lipid Metabolism and Gut Microbiota Composition in C57BL/6J Mice. *J Agric Food Chem* **65**, 6599–6607 (2017).
16. Djurasevic, S. *et al.* Beneficial Effect of Virgin Coconut Oil on Alloxan-Induced Diabetes and Microbiota Composition in Rats. *Plant Foods Hum Nutr* **73**, 295–301 (2018).
17. Ge, Y. *et al.* Effect of industrial trans-fatty acids-enriched diet on gut microbiota of C57BL/6 mice. *Eur J Nutr* **58**, 2625–2638 (2019).
18. Hua, Y. *et al.* Trans-fatty acids alter the gut microbiota in high-fat-diet-induced obese rats. *Br J Nutr* **124**, 1251–1263 (2020).
19. Hidalgo, M. *et al.* Changes in Gut Microbiota Linked to a Reduction in Systolic Blood Pressure in Spontaneously Hypertensive Rats Fed an Extra Virgin Olive Oil-Enriched Diet. *Plant Foods Hum Nutr* **73**, 1–6 (2018).
20. Mujico, J. R., Baccan, G. C., Gheorghe, A., Díaz, L. E. & Marcos, A. Changes in gut microbiota due to supplemented fatty acids in diet-induced obese mice. *Br J Nutr* **110**, 711–720 (2013).
21. Ghosh, S. *et al.* Fish oil attenuates omega-6 polyunsaturated fatty acid-induced dysbiosis and infectious colitis but impairs LPS dephosphorylation activity causing sepsis. *PLoS One* **8**, e55468 (2013).
22. Yu, H.-N. *et al.* Effects of fish oil with a high content of n-3 polyunsaturated fatty acids on mouse gut microbiota. *Arch Med Res* **45**, 195–202 (2014).

23. Caesar, R., Tremaroli, V., Kovatcheva-Datchary, P., Cani, P. D. & Bäckhed, F. Crosstalk between Gut Microbiota and Dietary Lipids Aggravates WAT Inflammation through TLR Signaling. *Cell Metab* **22**, 658–668 (2015).
24. Robertson, R. C. *et al.* Omega-3 polyunsaturated fatty acids critically regulate behaviour and gut microbiota development in adolescence and adulthood. *Brain Behav Immun* **59**, 21–37 (2017).
25. Lee, H.-C. *et al.* A high linoleic acid diet exacerbates metabolic responses and gut microbiota dysbiosis in obese rats with diabetes mellitus. *Food Funct* **10**, 786–798 (2019).
26. Patrone, V. *et al.* Short-term modifications in the distal gut microbiota of weaning mice induced by a high-fat diet. *Microbiology (Reading)* **158**, 983–992 (2012).
